# Refractory Autonomic Instability in Mild Traumatic Brain Injury: A Case Report

**DOI:** 10.7759/cureus.55634

**Published:** 2024-03-06

**Authors:** Rory J Siegel, Michael G Schloss, Jennifer Gray

**Affiliations:** 1 Physical Medicine and Rehabilitation, Mercy Medical Center, Catholic Health System, Rockville Center, USA; 2 Physical Medicine and Rehabilitation, Saint Charles Hospital and Rehab, Port Jefferson, USA

**Keywords:** concussion recovery, cardiac catheter ablation, autonomic nervous system dysfunction, brain concussion, post traumatic brain injury

## Abstract

Although the specific relationship between concussion and the autonomic nervous system (ANS) has not been fully elucidated, it is generally understood that the pathologic response after a traumatic brain injury (TBI) is linked with systolic cardiac dysfunction. In this case, we present a patient with multiple concussion injuries over a five-year period who exhibited severe cardiac and autonomic dysfunction, in addition to prolonged impairments in vestibular function, oculomotor function, cognitive function, and headaches.

The patient is a 28-year-old male with a past medical history of multiple concussions, with the first concussion occurring due to a skiing accident in January 2015. He initially presented in October 2016 after sustaining a concussion due to a motor vehicle accident (MVA) without loss of consciousness (LOC) two weeks prior. In July 2017, the patient was involved in another MVA with a positive head strike and without LOC, causing his third concussion. After each of his first three concussions, he displayed various symptoms that eventually resolved. In October 2020, the patient suffered a syncopal ground-level fall with several minutes of LOC due to dehydration and lightheadedness, leading to his fourth concussion. His fourth concussion resulted in chronic autonomic dysfunction with resting tachycardia refractory to medical management, and he eventually underwent a cardiac ablation. Although the patient underwent a cardiac ablation, his tachycardia and dysautonomia still cause dysfunction in his daily life. With millions of people living with the sequelae of TBI, the recognition and treatment of autonomic dysfunction should be a continued focus in brain injury research.

## Introduction

It is estimated that approximately 250,000 traumatic brain injury (TBI)-related hospitalizations occur every year and that there are between 3.2 and 5.3 million people in the United States living with TBI-related disabilities [1]. TBI can be further sub-classified into mild, moderate, or severe, with three out of every four TBI being documented as mild [2]. Concussions, per the International Conference on Concussion in Sport, are defined as "a complex pathophysiologic process affecting the brain, induced by traumatic biomechanical forces" [3]. Immediate symptoms can be physical, sensory and cognitive and often can include headache, nausea, vomiting, fatigue, sensitivity to light or sound, and a state of being disoriented or confused [4]. Other long-term associated conditions include post-concussion syndrome, persistent central nervous system neuropathies, psychological conditions or anxiety responses, pituitary dysfunction, vestibular dysfunction, and chronic pain [5]. Although the specific relationship between concussion and the autonomic nervous system (ANS) has not been fully elucidated, it is generally understood that the pathologic response after a TBI is linked with multiple-system organ dysfunction, specifically hemodynamically regulatory systems and cardiac dysfunction. Elevated intracranial pressure, inflammatory cytokines, cerebral vascular damage, and endothelial damage together have been conceptualized to be causal factors in disrupting the ANS homeostasis post-traumatic brain injury [6-8]. In this case, we present a patient with multiple concussion injuries over a five-year period who exhibited severe autonomic dysfunction in addition to prolonged impairments in vestibular function, oculomotor function, cognitive function, and headaches.

## Case presentation

A 28-year-old male with a past medical history of multiple concussions, with the first concussion occurring due to a skiing accident in January 2015 (Table 1). An incidental pontine capillary telangiectasia was discovered on the initial imaging workup at that time. He initially presented to our office on October 12, 2016, for an outpatient concussion evaluation after sustaining a subsequent concussion due to a motor vehicle accident (MVA) without loss of consciousness (LOC) two weeks prior. Computed tomography of the head demonstrated known capillary telangiectasia, unchanged from prior imaging.

**Table 1:** Timeline of events with recorded vitals and notable symptoms

Date	Heart rate (bpm)	Blood pressure (mmHg)	Notable symptoms
First concussion: January 2015 - skiing accident			
Second concussion: September 2016 - motor vehicle accident (MVA)			
October 12th, 2016	77	110/70	Vestibular dysfunction, cognitive difficulties, insomnia
Third concussion: July 21, 2017 - motor vehicle accident (MVA)			
August 14th, 2017	90	129/79	Sleep disturbance, visual difficulties
June 2019	99	134/84	Resolution of concussion-related symptoms
Fourth concussion: October 30, 2020 - ground level fall (syncopal event)			
November 2020	81	116/79	Fatigue, photophobia, nausea, vomiting, dizziness, headaches, decreased concentration, difficulty sleeping
December 2020	108	116/79	Consistent symptoms from prior visit with headaches
January-April 2021	120-136	136-138/81-92	Short-term memory difficulties, recurrent syncopal episodes with exercise testing
March 2021 - January 2022	94-115	60-86/96-144	Orthostatic hypotension leading to syncopal events
May 2022 - patient underwent a cardiac ablation			
Summer 2022	108	128/79	Decreased ability to focus, vestibular function impairments (improving)

Vital signs were obtained and included a heart rate of 77 beats per minute (bpm) and blood pressure of 110/70 mmHg. Primary concerns focused around vestibular dysfunction, difficulty performing cognitive tasks, and insomnia. In addition to activity modification, he was prescribed melatonin 3 milligrams (mg) as needed for sleep and instructed to start vestibular rehabilitation, which ultimately helped to resolve all concussion-related symptoms.

On July 21, 2017, the patient sustained a third concussion from an MVA striking his head but not sustaining LOC. While evaluated in the outpatient clinic on August 14, 2017, primary complaints centered around sleep disturbance and visual difficulties. Vital signs were obtained and showed a heart rate of 90 bpm and a blood pressure of 129/79 mmHg. The patient followed a similar low-stimulation treatment plan and obtained necessary follow-ups as ordered by our clinic. At follow-up in June 2019, all of his concussion-related symptoms were resolved. Vital signs included a heart rate of 99 bpm and a blood pressure of 134/84 mmHg.

On October 30, 2020, the patient suffered a syncopal ground-level fall with several minutes of LOC due to dehydration and lightheadedness in training for a law enforcement position. He had a delayed return home from his deployment and was seen in the office about one month following the injury, complaining of fatigue, photophobia, nausea, vomiting, dizziness, headaches, decreased concentration, and difficulty sleeping. Vital signs included a heart rate of 81 bpm and a blood pressure of 116/79 mmHg. The patient underwent magnetic resonance imaging (MRI) of the brain and cervical spine, which showed no new pathology. He was instructed to take naproxen 500 mg twice daily for five days, additional acetaminophen as needed for headaches, ondansetron as needed for nausea, and meclizine as needed for dizziness.

At his follow-up appointment two weeks later, the patient exhibited resting tachycardia. His heart rate (HR) was 108 bpm, and his blood pressure (BP) was 116/79 mmHg. Our office referred the patient to neurology and cardiology. The neurologist started the patient on venlafaxine 37.5 mg nightly and sumatriptan 100 mg as needed for headaches, and ordered an electroencephalogram (EEG) to rule out any underlying ictal events, which was unremarkable. Subsequent electrocardiogram (ECG) demonstrated sinus tachycardia, and initial transthoracic echocardiogram demonstrated a left-ventricular ejection fraction (LVEF) of 55-60% with mild tricuspid regurgitation and mild mitral regurgitation. Cardiology diagnosed the patient with dysautonomia from his head injury and recommended using a stationary bike daily as tolerated for light aerobic exercise. An exercise stress test performed in January 2021 was inconclusive as the patient had poor exercise tolerance secondary to hypotension. Over the next few months, during follow-up appointments at our clinic, his HR ranged from 120-136 bpm and BP 136-138/81-92 mmHg. He continued to endorse trouble with short-term memory and reported enduring recurrent syncopal episodes during the usage of a stationary bike. Our office prescribed a neuropsychologic evaluation along with guided meditation and acupuncture, and he underwent an endocrinology evaluation that ruled out a hypothalamic-pituitary-adrenal disorder. 

From March 2021 to January 2022, recorded vitals ranged from a heart rate of 94 to 115 bpm and blood pressure of 60-86/96-144 mmHg. The patient was treated with propranolol 20 mg twice daily to address the persistent tachycardia, transitioned to metoprolol succinate 25 g twice daily, and trialed on midodrine 5 mg twice daily before being discontinued after a syncopal event in January 2022. His cardiologist considered the diagnosis of postural orthostatic tachycardia syndrome (POTS), and Holter monitor placement demonstrated sinus rhythm with tachycardia roughly 25% of the recorded time in that period. EKG recorded at that time demonstrated sinus tachycardia without any other abnormalities. The diagnosis of POTS was later excluded after further evaluation of his persistent tachycardia despite positional changes. 

Ultimately, the patient underwent a cardiac ablation in May 2022 as he failed medical management in addressing his poorly tolerated episodes of tachycardia. Furthermore, the rationale being if he had a predisposition to a cardiogenic arrhythmia (i.e., sinus tachycardia), he would be more sensitive to the brain injury-induced dysautonomia. Post-procedure, the patient noted decreased palpitations and periods of tachycardia since the procedure but complained of episodes of intermittent bradycardia. His cognitive symptoms, including decreased ability to focus and impairments in vestibular function, continue to persist but are slowly improving. Vital signs at his most recent visit were a heart rate of 108 bpm and blood pressure of 128/79 mmHg.

## Discussion

Pathophysiology of dysautonomia

The ANS exerts involuntary control over the body's parasympathetic and sympathetic responses and is the main influence over cardiac responses toward environmental stressors. The parasympathetic system innervates the body towards a state of rest by lowering the heart rate and blood pressure, while the sympathetic system promotes a "fight or flight" response of increased heart rate and blood pressure [9]. Recent research has concluded that dysautonomia, or dysfunction of the ANS, significantly contributes to symptoms experienced after a concussion [10]. Our patient suffered significant resting tachycardia after his fourth concussion, likely due to a lack of vagal parasympathetic input that is consistent with dysautonomia.

A few previous studies have compared the incidence of dysautonomia with severe traumatic brain injuries or those with a Glasgow Coma Scale (GCS) less than or equal to eight on initial evaluation [11]. Incidence of dysautonomia within this specific population ranged from 10-20% [12]. Additionally, higher incidences were linked to those exhibiting spasticity or diffuse axonal injury, or shearing of the brain's nerve fibers in a fixed skull after an impact event [13]. The patient presented here, however, only suffered multiple mild traumatic brain injuries (defined as a GCS between 12 and 15), and he did not suffer symptoms of diaphoresis or dystonia, commonly found within patients with diffuse axonal injury [11, 13]. Although this patient underwent a prolonged rehabilitation secondary to dysautonomic symptoms, the case presented here warrants more investigation of the causal relationship between multiple mild brain injuries and the autonomic system.

Workup and treatment

Over the past decade, heart rate variability (HRV) has become a reliable measure of cardiac autonomic function. Furthermore, many recent studies have attempted to use HRV as a quantifiable method to investigate the functionality of the ANS in post-concussive patients. HRV accounts for the "beat-to-beat" alterations in one's heart rate at a resting state and, therefore, reflects the inhibitory control of the parasympathetic nervous system on the sinoatrial node [14]. When responding to environmental stressors, greater HRV signifies an adequate response of the ANS [15]. In turn, a lesser or reduced HRV signifies an inadequate response to stressors and a more pronounced imbalance between parasympathetic and sympathetic input. Reduced HRV in response to stressors has been found to be associated with the development of cardiovascular disease, inflammation, obesity, and psychiatric disorders [16]. However, at rest and without the presence of a perceived stressor, a greater HRV indicates a lack of parasympathetic control over the sympathetic nervous system. This leads to hyperarousal, as the body remains in a continual sympathetically-mediated state [17]. One preliminary study of HRV completed in 2021 demonstrated an association with learning, memory, and attention in patients during the post-acute phase of concussion injury [15]. A greater HRV at rest was noted in patients with more severe emotional and cognitive symptoms. Additionally, a greater HRV at rest was indicative of worsened performance on cognitive and recall tasks (Groton Maze Learning, One-Back, and Groton Maze Delayed-Recall), with acceptable validity and reliability [15].

Radiofrequency catheter cardiac ablation has become a treatment modality adopted by many cardiologists and electrophysiologists when medical management is unsuccessful at addressing tachyarrhythmias [18]. The three primary indications warranting a cardiac ablation include symptomatic idiopathic ventricular tachycardia, atrial fibrillation that is non-responsive to medical management and symptomatic supraventricular tachycardia secondary to atrioventricular reentrant tachycardia (AVRT), atrioventricular nodal reentrant tachycardia (AVNRT), unifocal atrial tachycardia or atrial flutter [18]. However, because of its low side effect profile, cardiac ablation is now the treatment option of choice for many electrophysiologists when facing tachyarrhythmia [18]. Notable side effects include vascular complications (2-4%), cardiac trauma including tamponade (1-2%), heart block (0.5%), and death, MI, or stroke (0.05%-0.01%). Although the success rate is greater than 90% for AVRT and AVNRTs, the statistics are not as promising for symptomatic inappropriate sinus tachycardia, which was experienced by this patient. The success rate is 86.4%, with a recurrence rate of 19.6%, according to a systematic review published in 2019 [19]. After the cardiac ablation performed on our patient in May of 2022, his documented heart rate ranged from 105-108 bpm over the next three months. Dysautonomia continues to significantly impact the patient, albeit with minor subjective improvement, since the ablation.

One exciting novel potential method of treatment for dysautonomia is transcutaneous vagal nerve stimulation (tVNS). tVNS acts primarily via the nucleus tractus solitarius (NTS), which is involved in emotional regulation sites throughout the brain to inhibit fight or flight responses [17]. Increased activity of the NTS, therefore, combats a hyperarousal state by increasing vagal tone. A pioneering study by Lamb et al. demonstrated the benefits of transcutaneous vagal nerve stimulation for augmenting parasympathetic activity and decreasing sympathetic activity at rest among post-traumatic stress disorder (PTSD) patients [17]. Although this patient does not have PTSD, he has similarly documented dysfunction of his ANS and chronic stress that could lead to a similar pathologic state. Another study investigating tVNS on the ANS by Tobaldini et al. demonstrated that vagal stimulation can effectively lower both central and peripheral sympathetic tone [20]. Application of tVNS for a total of 10 minutes in a supine position and 15 minutes standing upright resulted in a greater than 4% decrease in resting heart rate in healthy subjects [20]. The results of this study show a direct neuro-modulator effect on the cardiovascular system and highlight the potential benefit of tVNS for addressing drug-refractory dysautonomia [20]. Although more studies are needed to confirm the specific and isolated effects tVNS has on the ANS, the safety profile and feasibility of this treatment modality greatly surpasses other more invasive measures. Adding tVNS to our patient's treatment plan could potentially provide significant relief from his refractory dysautonomia caused by multiple mild traumatic brain injuries.

## Conclusions

Continued assessment of this patient’s vital signs, cardiac function, and response to external stressors will be fundamental in providing him with proper long-term treatment. Despite undergoing cardiac ablation, his tachycardia and dysautonomia still appear to be causing dysfunction in his daily life. Transcutaneous vagal nerve stimulation is a non-invasive treatment method that could potentially help mitigate the long-term cardiologic and neurocognitive effects of chronic stress on the body. Coupled with established medical management, tVNS in concussion patients could potentially reduce the need for operative measures such as cardiac ablation and is an avenue of interest. With millions of TBI patients in today’s society, better management of autonomic stability needs to become a focus for all survivors.
